# Leveraging Assistive Technology Resources to Support Aging in Place: A Scoping Study

**DOI:** 10.1093/geroni/igaa057.330

**Published:** 2020-12-16

**Authors:** George Mois, Jenay Beer

**Affiliations:** 1 University of Georgia, Lawrenceville, Georgia, United States; 2 Institute of Gerontology, Athens, Georgia, United States

## Abstract

Aging in place is the preferred living arrangement for most older adults. However, the challenges that often accompany longevity coupled with housing which lacks proper modifications presents concerns about older adults’ safety and wellbeing. Advancements in assistive technologies have promising potential in helping address many of these challenges and support aging in place. The purpose of this scoping review was to survey the current literature to understand why, how, and what assistive technologies are adopted and utilized to help support aging in place. We followed the Arksey and O’Malley (2005) methodological framework for scoping studies, searching seven databases and systematically assessed 611 titles/articles. Findings were organized using frequencies and themes. Following the inclusion/exclusion criteria, 12 articles were included. Upon thematic analysis, three main themes emerged: 1.) challenges experienced in the context of aging in place, 2.) technology adoption, and 3.) technology types and applications. Findings indicate technology can serve an important role in helping support aging in place and can serve as a medium to deliver and increase access to resources to support physical, social, and psychological wellbeing. The technologies most frequently utilized include personal devices and smart home technologies. The adoption and use of technologies can be impacted by the perceived ease of use, perceived usability, family/caregiver, self-selection, involvement in technology development, policies supporting access, and environment factors. Our findings indicate that there is a current gap in the understanding of how older adults are interacting with technology and how long term use impacts wellbeing and aging in place.

